# Zrsr2 Is Essential for the Embryonic Development and Splicing of Minor Introns in RNA and Protein Processing Genes in Zebrafish

**DOI:** 10.3390/ijms231810668

**Published:** 2022-09-14

**Authors:** Rachel Weinstein, Kevin Bishop, Elizabeth Broadbridge, Kai Yu, Blake Carrington, Abdel Elkahloun, Tao Zhen, Wuhong Pei, Shawn M. Burgess, Paul Liu, Erica Bresciani, Raman Sood

**Affiliations:** 1 Zebrafish Core, Translational and Functional Genomics Branch, National Human Genome Research Institute, National Institutes of Health, Bethesda, MD 20892, USA; 2 Oncogenesis and Development Section, Translational and Functional Genomics Branch, National Human Genome Research Institute, National Institutes of Health, Bethesda, MD 20892, USA; 3 Microarray Core, Cancer Genetics and Comparative Genomics Branch, National Human Genome Research Institute, National Institutes of Health, Bethesda, MD 20892, USA; 4 Developmental Genomics Section, Translational and Functional Genomics Branch, National Human Genome Research Institute, National Institutes of Health, Bethesda, MD 20892, USA

**Keywords:** ZRSR2, U12 introns, intron retention, zebrafish, CRISPR/Cas9

## Abstract

ZRSR2 (zinc finger CCCH-type, RNA binding motif and serine/arginine rich 2) is an essential splicing factor involved in 3′ splice-site recognition as a component of both the major and minor spliceosomes that mediate the splicing of U2-type (major) and U12-type (minor) introns, respectively. Studies of *ZRSR2*-depleted cell lines and *ZRSR2*-mutated patient samples revealed its essential role in the U12-dependent minor spliceosome. However, the role of *ZRSR2* during embryonic development is not clear, as its function is compensated for by *Zrsr1* in mice. Here, we utilized the zebrafish model to investigate the role of *zrsr2* during embryonic development. Using CRISPR/Cas9 technology, we generated a *zrsr2*-knockout zebrafish line, termed *zrsr2^hg129/hg129^* (p.Trp167Argfs*9) and examined embryo development in the homozygous mutant embryos. *zrsr2^hg129/hg129^* embryos displayed multiple developmental defects starting at 4 days post fertilization (dpf) and died after 8 dpf, suggesting that proper Zrsr2 function is required during embryonic development. The global transcriptome analysis of 3 dpf *zrsr2^hg129/hg129^* embryos revealed that the loss of Zrsr2 results in the downregulation of essential metabolic pathways and the aberrant retention of minor introns in about one-third of all minor intron-containing genes in zebrafish. Overall, our study has demonstrated that the role of Zrsr2 as a component of the minor spliceosome is conserved and critical for proper embryonic development in zebrafish.

## 1. Introduction

ZRSR2 (zinc finger CCCH-type, RNA binding motif and serine/arginine rich 2) is an essential splicing factor involved in the recognition of 3′ splice sites during pre-mRNA splicing [1,2]. Pre-mRNA splicing is carried out by the major and minor spliceosomes, which target U2- and U12-type introns, respectively [3,4]. These two types of introns differ in their distinct splice site and branch point sequences [5,6]. Most introns (>99%) in a eukaryotic genome are of a U2-type, while U12-type introns represent <0.5% of the total introns [7,8]. Thus, U2-type and U12-type introns are also termed major and minor introns, respectively. Interestingly, all organisms with minor introns in their genome carry an ortholog of *ZRSR2*, suggesting its conserved role in their splicing [1].

The human *ZRSR2* gene is localized on the X chromosome, and somatic loss-of-function mutations in *ZRSR2* are reported in ∼5% of patients with myelodysplastic syndrome (MDS) [9,10,11,12,13,14]. In addition, *ZRSR2* mutations are occasionally associated with other hematological diseases, i.e., chronic myelomonocytic leukemia, refractory anemia with ringed sideroblasts, secondary acute myeloid leukemia, and blastic plasmacytoid dendritic cell neoplasm [15,16,17,18]. Transcriptome analyses of *ZRSR2*-depleted cells and bone marrow samples from MDS patients with *ZRSR2* mutations revealed an increase in retention of U12-type introns, while U2-type introns remained largely unaffected [19,20,21]. These findings provide strong support for the essential role of ZRSR2 in the minor spliceosome [19,20,21].

Recently, multiple research groups have investigated the in vivo functions of ZRSR2 using either traditional or conditional knockout mouse models [21,22,23,24]. These studies showed that a functional paralog *Zrsr1* compensates for the loss of *Zrsr2* in mice [23,24]. *ZRSR1* in humans is considered a non-functional pseudogene (*ZRSR2P1*), but it is functional in mice [22,23,24,25]. While homozygous mutant mice for either *Zrsr1* or *Zrsr2* developed normally and only exhibited specific phenotypes during adulthood, double knockout mice were embryonic lethal and showed an arrest in embryo development at the 2-cell stage [22,23,24,25]. Thus, studies of these mouse models were unable to reveal specific roles of Zrsr2 in vertebrate embryonic development [21,22,23,24]. Zebrafish are an alternate robust vertebrate model and are suitable for studying the role of genes in embryonic development due to their rapid and external embryonic development and optically clear embryos [26,27,28]. Furthermore, based on the currently available genome sequence, there is no evidence for a *Zrsr1* ortholog, and there is a single *zrsr2* ortholog in the zebrafish genome. Thus, in this study, we utilized the zebrafish model to investigate the specific roles of *zrsr2* in embryonic development.

Here, we report our work on the analysis of *zrsr2* expression and the effect of its loss of function during embryonic development. Our data showed that Zrsr2 is essential for multiple processes, including hematopoiesis during embryonic development and its role in the splicing of U12-type introns as a component of the minor spliceosome is conserved in zebrafish. More than one-third of all zebrafish genes that contain U12-type introns (221/612) are dysregulated in the absence of Zrsr2 due to intron retention (IR). Interestingly, a vast majority of these dysregulated genes are involved in RNA and protein processing, thus implicating the importance of these pathways in embryonic development.

## 2. Results

### 2.1. The Zebrafish Zrsr2 Protein Has All the Functional Domains of Human ZRSR2 and an Extra C-Terminal Region

**Figure 1:**
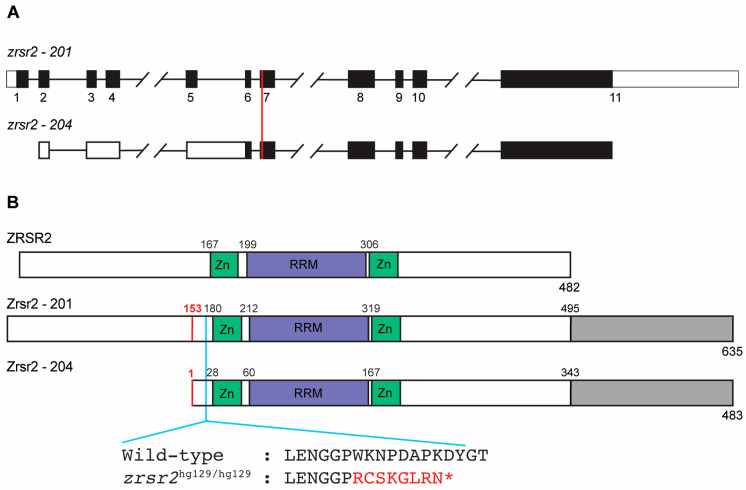
Genomic organization and functional domains of zebrafish Zrsr2. (**A**) A schematic of the genomic structures of *zrsr2* isoforms 201 and 204, with exons shown as rectangles and introns as lines connecting the exons. Filled rectangles mark the coding sequence, and white rectangles mark the untranslated regions. The vertical red line marks the genomic target region of the sgRNA used for CRISPR/Cas9 knockout. (**B**) The alignment of the human ZRSR2 protein with isoforms 201 and 204 of zebrafish Zrsr2, showing the location of conserved functional domains: Zn = Zinc finger domains, RRM = RNA recognition motif. Gray color marks the extra C-terminal region in zebrafish Zrsr2. Black numbers indicate the amino acid number for the start of each domain. Red numbers mark the methionine at position 153 in isoform 201, which is the translation start site of Zrsr2-204. Blue line marks the location of the CRISPR/Cas9-induced mutation and partial amino acid sequences of WT and mutant proteins, with red color marking the frameshift and premature termination codon marked as *.

The zebrafish genome contains a single copy of the *zrsr2* gene localized on chromosome 11. It encodes two major isoforms, isoform 201 (635 amino acids in length; GenBank accession number: XM_021479948) and isoform 204 (483 amino acids in length; GenBank accession number: XM_021479949). Isoform 204 has a longer 5′ untranslated region due to alternate splicing between exons 3 to 6, and its translation start site lies in exon 6 (Figure 1A). Thus, the protein encoded by isoform 204 lacks the first 152 amino acids but is otherwise identical to isoform 201 (Figure 1B). The major ZRSR2 isoform in humans is isoform 201 which contains all known functional domains (482 amino acids in length; GenBank accession number: NM_005089). The alignment of both zebrafish isoforms with human ZRSR2-201 showed an overall protein similarity of 58%, with a higher level of similarity between the functional domains (Figure 1B, Supplementary Figure S1). Interestingly, there is an extra sequence of 140 amino acids at the C-terminus of the zebrafish Zrsr2 compared to the human ZRSR2 (Figure 1B, Supplementary Figure S1). Analysis of this extra C-terminus part by BLASTp (https://blast.ncbi.nlm.nih.gov/ accessed on 21 October 2019) showed that it is also present in the ZRSR2 orthologs from other species of fish. However, this region did not show any recognizable functional domains.

### 2.2. Both Isoforms of zrsr2 Are Ubiquitously Expressed during Embryonic Development

**Figure 2:**
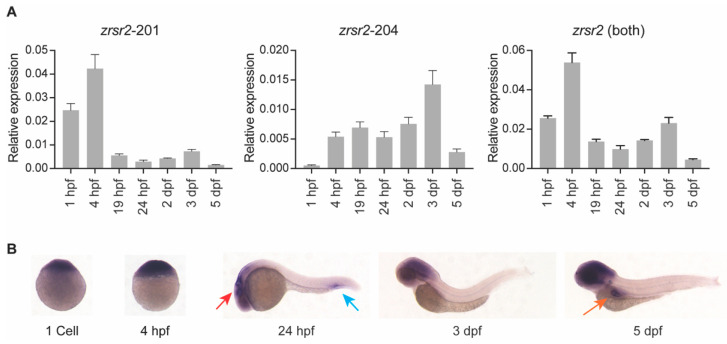
*zrsr2* expression during zebrafish embryonic development. (**A**) qRT-PCR data showing expression of *zrsr2* isoforms using primers unique to each isoform, and total expression using primers common to both isoforms in WT embryos from a series of stages during development as marked across the bottom of each plot. (**B**) WISH with *zrsr2* antisense probe, showing its sites of expression in WT embryos at 1 cell, 4 hpf, 24 hpf, 3 dpf, and 5 dpf as marked. Specific *zrsr2* expression is seen in the developing brain (red arrow) and the peripheral blood island (blue arrow) at 24 hpf, and in the intestinal bulb (orange arrow) at 5 dpf.

To understand the role of *zrsr2* in normal embryonic development, we examined the spatial and temporal patterns of the expression of *zrsr2* by RT-PCR, quantitative RT-PCR (qRT-PCR), and whole mount in situ hybridization (WISH) in wild-type (WT) embryos from a series of developmental stages. RT-PCR and qRT-PCR were performed using primers unique to the shared regions of isoforms 201 and 204 (Supplementary Table S1). Overall, the traditional and quantitative RT-PCR data were consistent with each other and revealed that *zrsr2* is expressed throughout embryonic development, with some variation in the levels of expression of isoforms 201 and 204 at specific stages (Figure 2A, Supplementary Figure S2). Interestingly, the data using isoform-specific primers revealed that isoform 201 is maternally inherited, while the expression of isoform 204 begins after the start of zygotic transcription (Figure 2A, Supplementary Figure S2). The RT-PCR product from the region shared by both isoforms (Supplementary Figure S2) was cloned to generate a *zrsr2* riboprobe for WISH (Figure 2B). Our data revealed that, subsequent to the ubiquitous expression at the beginning of zygotic transcription, *zrsr2* expression is enriched in the anterior region of the embryo, specifically in the brain from after 24 h post-fertilization (hpf) to 5 dpf (Figure 2B). These data are consistent with the findings from a mouse model in that a large proportion of U12-type intron-containing genes are expressed in the brain [29]. Specific *zrsr2* expression was also observed in the ventral side of the tail at 24 hpf and in the intestinal bulb at 5 dpf (Figure 2B). Overall, our WISH data suggested that, in addition to being ubiquitously expressed as an essential splicing factor, *zrsr2* is highly expressed in certain developing tissues, which may indicate regions that are actively growing and, thus, more dependent on the splicing machinery.

### 2.3. zrsr2 Knockout Results in Abnormal Embryonic Development, Hematopoietic Defects, and Lethality by 8 dpf

To understand the in vivo functions of Zrsr2 during vertebrate development, we used CRISPR/Cas9 technology to generate a zebrafish *zrsr2*-knockout model. To ensure a complete loss-of-function model, a single guide RNA was designed to target exon 7, which is shared by both isoforms (Figure 1A) and is upstream of all known functional domains (Figure 1B). We selected a mutant allele with an 11 bp deletion (c.495delACCCTGGAAAA; p.Trp167Argfs*9), which leads to loss of all functional domains due to a frameshift followed by premature truncation (Figure 1B). The RT-PCR and sequencing of WT and the homozygous mutant embryos confirmed that the mutant allele was expressed, and the deletion did not cause any detectable splicing defects in the *zrsr2* transcript (Supplementary Figure S3A). This mutant zebrafish line, referred to as *zrsr2^hg129/hg129^* in subsequent sections, provided a robust knockout model for the investigation of the in vivo functions of Zrsr2 during vertebrate development.

**Figure 3:**
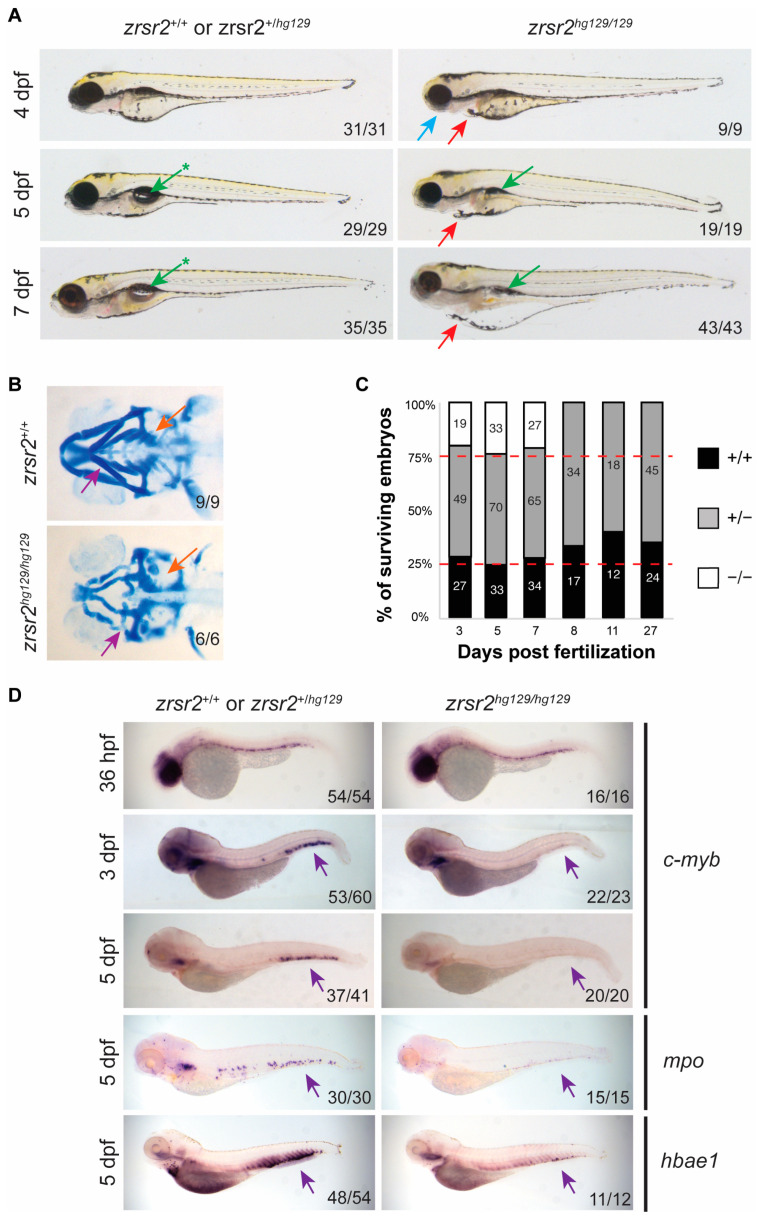
*zrsr2*-knockout embryos display morphological and hematopoietic defects. (**A**) Morphological phenotypes of *zrsr2^hg129/hg129^* embryos at 4, 5, and 7 dpf are shown in the right panel compared to their WT siblings in the left panel. Blue arrow marks defective jaw, red arrows mark cardiac edema, green arrows mark uninflated swim bladder, and green arrows with asterisks mark the normal inflated swim bladder in WT embryos. All images depict lateral views of the embryos. (**B**) Ventral view of embryos stained with Alcian blue depicting the cartilage of developing jaw of WT embryos (top panel) and *zrsr2^hg129/hg129^* embryos (bottom panel) at 4 dpf with purple arrows marking the mandibular cartilage and orange arrows marking the pharyngeal arches. (**C**) Survival of embryos collected from adult *zrsr2^hg129/+^* in-cross. The bar graph displays the genotype ratios as a percentage of total sampled embryos, and red lines indicate the expected Mendelian offspring ratios (1:2:1; *zrsr2^+/+^*: *zrsr2^hg129/+^*: *zrsr2^hg129/hg129^*). Numbers in the bars represent the number of embryos of each genotype. Clutches were sampled for genotype makeup at multiple time points as marked on the X-axis. *zrsr2^hg129/hg129^* embryos died by 8 dpf, while *zrsr2^hg129/+^* and *zrsr2^+/+^* embryos survived to adulthood. (**D**) WISH for definitive hematopoietic markers c-*myb* (36 hpf, 3 dpf, and 5 dpf), *mpo* (5 dpf) and *hbae1* (5 dpf), showing expression in *zrsr2^hg129/hg129^* embryos (right panel) compared to their siblings (left panel). Lateral views of embryos are shown in all images, with purple arrows marking the CHT region, showing reduced expression in *zrsr2^hg129/hg129^* embryos.

To determine the effect that loss of Zrsr2 function had on embryonic development, we observed the morphological phenotypes of embryos obtained from pairwise crosses of heterozygous fish for several days after fertilization. The *zrsr2^hg129/hg129^* embryos displayed normal morphology for the first three days of development and normal expression of the markers of early development, such as *kdrl*, *myod1,* and *shh* (Supplementary Figure S3B). At 4 dpf, the *zrsr2^hg129/hg129^* embryos could be distinguished from their WT and heterozygous siblings by a smaller head size, lack of a proper jaw, and mild (∼75% of mutant embryos) to moderate (∼25% of mutant embryos) cardiac edema (Figure 3A). Further analysis of the jaw region by Alcian blue staining revealed defects in the mandibular cartilage and pharyngeal arches in *zrsr2^hg129/hg129^* embryos (Figure 3B). Between 5 dpf and 7 dpf, the cardiac edema and jaw defects became more pronounced, and the swim bladder failed to inflate in the mutant embryos (Figure 3A). All *zrsr2^hg129/hg129^* embryos died by 8 dpf (Figure 3C). These findings suggested that Zrsr2 is essential for multiple processes during embryonic development and the normal development of *zrsr2^hg129/hg129^* embryos for the first 3 dpf is most likely due to the compensation by maternally supplied *zrsr2* mRNA.

Since recurrent ZRSR2 somatic mutations are found in hematological diseases, we investigated the role of Zrsr2 in hematopoiesis ontogeny by WISH with lineage-specific markers for both primitive and definitive hematopoiesis [30,31]. The onset of primitive hematopoiesis was normal in *zrsr2*-knockout embryos, as indicated by the normal levels of expression of erythroid and myeloid markers, *gata1*, *scl*, *l-plastin,* and *mpo*, at 14 to 24 hpf (Supplementary Figures S4A–C). Interestingly, *zrsr2^hg129/hg129^* embryos displayed mild anemia at 2 dpf, as indicated by a significantly reduced *o*-dianisidine staining compared to their siblings (Supplementary Figure S4D–E). Expression of the hematopoietic stem cell (HSC) markers, *runx1* and *c-myb*, showed that the emergence of HSCs is normal in the mutant embryos (Figure 3D, Supplementary Figure S5). However, no *c-myb* expression was detected in the caudal hematopoietic tissue (CHT) region in 3 and 5 dpf mutant embryos (Figure 3D). The lack of HSCs in the CHT region is accompanied by a subsequent reduction in the cells of the erythroid (*hbae1*) and myeloid (*mpo*) lineages at 5 dpf (Figure 3D). These data suggested that Zrsr2 is important for the maintenance of HSCs during definitive hematopoiesis.

To confirm that the observed phenotypes in our mutant line are specific to the loss of Zrsr2 as opposed to being an off-target effect of CRISPR/Cas9 targeting, we used the crispant strategy, in which mosaic-injected embryos, with phenotypic penetrance based on the frequency of induced indels, are evaluated [32]. An independent sgRNA was designed to target exon 8, and the injected embryos were observed for morphological phenotypes using the original sgRNA as control. As expected, the crispant embryos for both sgRNAs recapitulated the morphological phenotypes observed in the germline mutants (Supplementary Figure S6A). Furthermore, similar to the *zrsr2^hg129/hg129^* embryos, crispants for both sgRNAs displayed a reduced expression of *c-myb* in the CHT at 3 dpf (Supplementary Figure S6B). Combining these data with our subsequent observations of *zrsr2^hg129/hg129^* embryos for up to five generations, consistently showing the phenotype-to-genotype correlations, we feel confident that our *zrsr2^hg129/hg129^* line is a robust model for the analysis of Zrsr2 deficiency. Overall, our data revealed that Zrsr2 is essential for multiple processes, including hematopoiesis ontogeny during embryonic development in zebrafish.

### 2.4. Zrsr2 Deficiency Leads to Perturbations in Metabolic Processes

**Figure 4:**
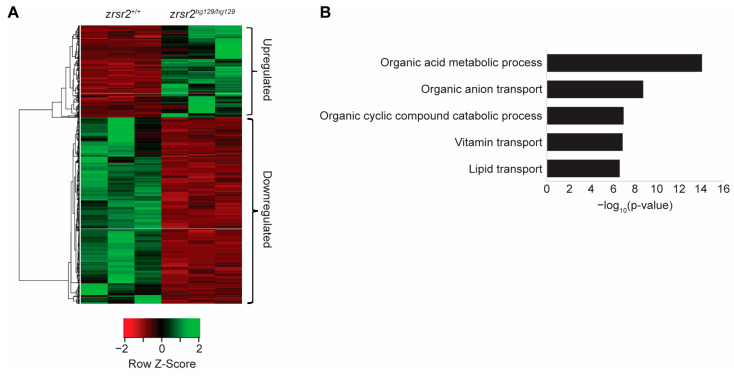
Loss of *zrsr2* leads to global gene expression changes in metabolic processes. (**A**) Heatmap of the differentially expressed genes in *zrsr2^hg129/hg129^* embryos compared to their WT controls at 3 dpf, with the color key shown below the heatmap. An amount of 455 genes were upregulated, and 917 genes were downregulated in the *zrsr2^hg129/hg129^* samples (fold change ≥ 2, *p* < 0.05). (**B**) Bar graph of the top 5 biological processes enriched among 917 downregulated genes in *zrsr2^hg129/hg129^* embryos at 3 dpf, with enrichment visualized by −log10 (*p*-value). Metabolic processes and transport of ions, vitamins, and lipids are among the most significantly enriched pathways among the downregulated genes.

We used RNA sequencing (RNA-Seq) from 3 dpf *zrsr2^hg129/hg129^* embryos before the onset of gross morphological defects to identify the biological processes affected by the loss of Zrsr2 in zebrafish. RNA-Seq was performed in triplicates from WT (*zrsr2^+/+^* embryos) and mutant (*zrsr2^hg129/hg129^*) embryos. All raw data were judged to be of good quality by their similar distributions of read counts, genomic coverage, and number of mappable reads in all six samples, indicating there were no outlier samples (Supplementary Figure S7A,B, Supplementary Table S2). RNA-Seq data revealed the reduced expression of both *zrsr2* isoforms, indicating a premature stop-codon mediated mRNA decay in the mutant embryos (Supplementary Figure S7C). Analysis of the RNA-Seq data with DESeq2 [33], using a fold change cut-off of 2 and p-adjusted value cutoff of 0.05, identified 1372 differentially expressed genes (DEGs) between *zrsr2^hg129/hg129^* and WT embryos (Figure 4A, Supplementary Table S3). Of these DEGs, 455 genes were upregulated, and 917 genes were downregulated in the *zrsr2^hg129/hg129^* embryos compared to the WT embryos (Supplementary Table S3). Interestingly, gene ontology (GO) analysis of the upregulated genes did not identify any specific biological processes to be significantly affected by the loss of Zrsr2, whereas the downregulated genes affected the metabolic processes, including the transport of vitamins and lipids (Figure 4B). Consistent with our data, widespread changes in metabolic pathways were observed in a *Drosophila* model with another disrupted minor splicing component, U6atac snRNA [34]. Overall, our data showed that the global transcriptomic changes caused by the Zrsr2 deficiency affected the general metabolic pathways essential for the proper development and homeostasis of embryonic tissues.

### 2.5. Loss of Zrsr2 Leads to the Preferential Retention of Minor Introns in Genes Involved in RNA and Protein Processing

As a component of the minor spliceosome, ZRSR2 is known to be essential for the splicing of the U12-type of introns [1]. Its loss of function in other biological models is associated with an increase in the retention of U12-type introns [19,21,23,24,35]. Therefore, we analyzed our RNA-Seq data for IR events with IRFinder [36]. We identified 284 differential IR events between *zrsr2^hg129/hg129^* and WT embryos (Supplementary Table S4). These 284 introns led to the abnormal splicing of 259 unique genes, as more than one intron was retained in some genes (Supplementary Table S4).

**Figure 5:**
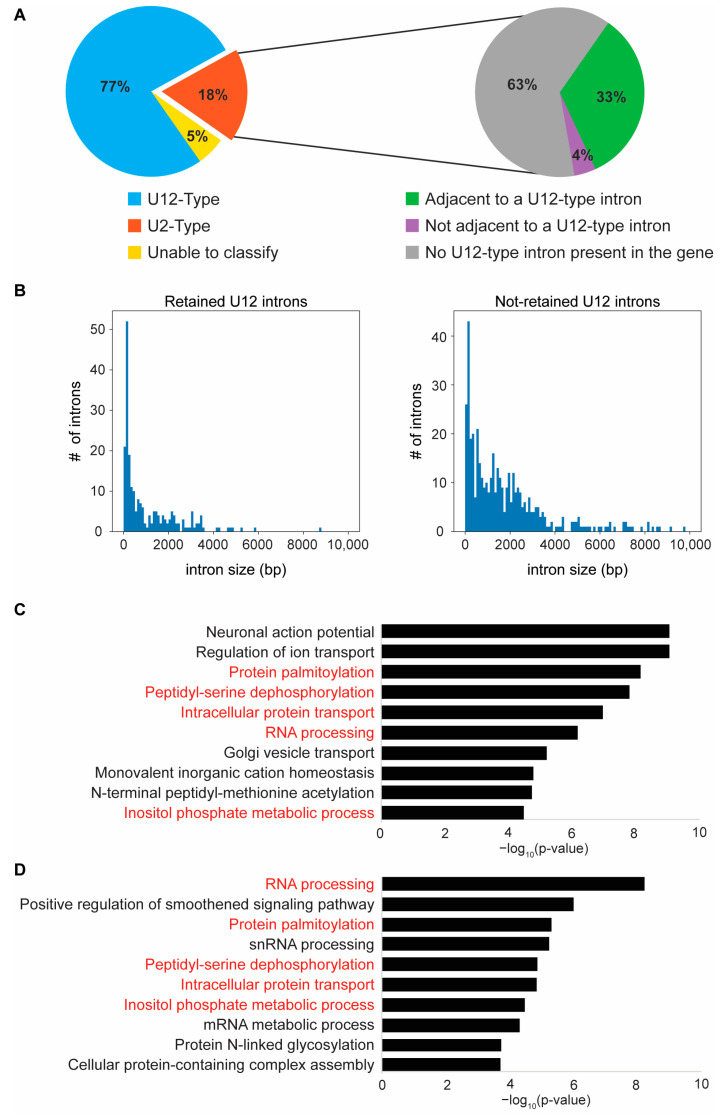
Loss of *zrsr2* leads to the differential retention of U12-type introns in RNA and protein processing genes. (**A**) Pie charts depicting the classifications for all differentially retained introns between *zrsr2^hg129/hg129^* and WT embryos into U12-type (blue) and U2-type (orange) introns (left) and the further classification of the retained U2-type introns (right) by whether they are adjacent to a known U12-type intron (green) or not adjacent but in a gene with a U12-type intron (purple), or in a gene with no known U12-type introns (grey). Some of the retained introns could not be classified into either category (yellow). (**B**) Plots of intron size distribution in the retained U12-type introns (left) and the non-retained U12-type introns (right). (**C**) The top 10 biological processes enriched in all genes (612) containing U12-type introns in the zebrafish genome according to IAOD. (**D**) The top 10 biological processes enriched in the set of U12-type intron containing genes (221) that have a differential IR between WT and *zrsr2^hg129/hg129^* embryos at 3 dpf, with enrichment visualized by −log10 (*p*-value). Five of the ten top pathways (written in red) are common in both gene sets (**C**,**D**).

Next, we used “The Intron Annotation and Orthology Database” (IAOD) [7] to determine if the retained introns were of the U2- or U12- subtypes. As reported previously in ZRSR2-depleted human and mouse models [19,20,21,24], most of the retained introns (218/284) in the *zrsr2^hg129/hg129^* mutant zebrafish were of the U12-type (Figure 5A). In addition, 19 out of 51 of the preferentially retained U2-type introns were found in those genes that also contained a U12-type intron (Figure 5A). Among the genes with retention of more than one intron, 15 genes showed the retention of both U2- and U12-type introns (Supplementary Table S4). Combining all these data, we found that, out of the 259 genes affected by IR in *zrsr2^hg129/hg129^* mutants, 221 genes contained the U12-type introns (Supplementary Table S4). Thus, our data provide strong support for the notion that the role of Zrsr2 in the splicing of U12-type introns is conserved in zebrafish.

A recent study [7] has identified a total of 662 U12-type introns that occur in 612 unique genes in zebrafish. Analysis of our RNA-Seq data showed that the majority of these U12-intron-containing genes are expressed in our dataset. Therefore, to understand why only a subset of all U12-type introns are retained (218 out of 662) in *zrsr2* mutants, we compared their sequence features with those not showing significant retention (444 out of 662). Our analysis revealed that while there were no differences in their nucleotide composition, the distribution of the GT-AG versus AT-AC splice sites, or sequence features around both splice sites, showed that the retained introns appeared to be smaller in size (Mean 1077 bp, Median 451 bp) compared to the non-retained introns (Mean 3556 bp, Median 1398 bp) (Figure 5B, Supplementary Figure S8, Supplementary Table S5). These data are consistent with previous findings [19] and suggest that the splicing of smaller U12-type introns is more sensitive to the loss of Zrsr2. To determine if the retention of minor introns affected the expression of corresponding genes, we queried our list of DEGs (Supplementary Table S3) for overlap with those genes showing retention of minor introns (Supplementary Table S4). Interestingly, only 43 genes (26 up, 17 down) showed the retention of minor introns and differential expression in the absence of Zrsr2 function. A similar comparison between genes without minor intron retention and differential expression identified 57 genes (29 up, 28 down). These findings indicate that the global changes in gene expression detected in *zrsr2^hg129/hg129^* mutants are not directly due to changes in the expression of the genes that contain U12-type introns but could represent the downstream effect of their aberrant splicing.

To identify the biological processes that are affected by the retention of the minor introns, we first identified all biological processes that require a properly functioning minor spliceosome by GO analysis of all U12-type intron-containing genes (612) in zebrafish (Figure 5C). Similar to the observations in other organisms [3,5], U12-type introns are present in the genes involved in neuronal action potential, ion transport, protein processing and transport, and RNA processing in zebrafish (Figure 5C). In comparison, the GO analysis of the 221 U12-intron-containing genes with differential IR in *zrsr2^hg129/hg129^* mutants (Figure 5D) showed that 5 of the top 10 biological processes that require the minor spliceosome were affected by the Zrsr2 deficiency, particularly those genes involved in RNA and protein processing pathways (Figure 5C,D). We found that, in the gene set with the retained introns, 48 genes were involved in RNA processing (i.e., RNA binding, RNA modification, RNA splicing, regulation of mRNA processing, RNA metabolic process, etc.), and 46 genes are involved in protein processing (i.e., protein modification by glycosylation, acetylation, phosphorylation, palmitoylation, regulation of protein stability by ubiquitination, deubiquitination, proteolysis and degradation, etc.) (Supplementary Table S4). Thus, our data suggest that Zrsr2 is specifically required for the regulation of genes involved in RNA and protein processing pathways during embryonic development diseases.

## 3. Discussion

ZRSR2 is an essential component of both the major and minor spliceosomes and is frequently mutated in hematopoietic disorders, particularly MDS. Recent studies have uncovered its requirement in the proper splicing of U12-type introns as a component of the minor spliceosome [19,20,21]. However, its role during vertebrate embryonic development is not known. In this study, we took advantage of the zebrafish model to study the impact of Zrsr2 deficiency on vertebrate embryonic development. We also showed that role of ZRSR2 in the splicing of the U12-type introns is conserved in zebrafish.

It has been suggested that minor introns are evolutionarily conserved as a mechanism for the regulation of cellular differentiation and multicellular growth [37]. The tissue-specific expression of genes with U12-type introns is regulated by their alternative splicing in both mice and humans [29]. Consistent with these findings, we observed that *zrsr2* is expressed throughout early zebrafish development when the cells are rapidly dividing and differentiating. The major isoform (*zrsr2*-201) is both maternally inherited and zygotically expressed throughout embryogenesis, while an alternate isoform (*zrsr2*-204) is expressed after the start of zygotic transcription. Our data on their differential expression during development suggests that the proteins encoded by each isoform perform specific functions which can be explored further using isoform-specific antibodies. *zrsr2* is highly expressed in the anterior region of the embryos and intestinal bulb, as has also been reported for other components of the minor spliceosome, e.g., *dhx15* and *rnpc3* [38,39]. The Zrsr2-deficient embryos developed normally for the first 3 days after fertilization, likely due to the presence of maternally-supplied WT *zrsr2* mRNA. At 4 dpf, they started to display pleiotropic phenotypes, indicating the requirement of proper Zrsr2 function in several developing tissues. It is possible that these tissues are more dependent on the minor spliceosome to produce appropriate mRNA transcripts for their growth and differentiation. Similar to the embryonic lethality observed upon disruption of the other components of the minor spliceosome in *Drosophila* and zebrafish [34,38,39], *zrsr2*-knockout fish displayed lethality by 8 dpf. Thus, our study adds to the growing evidence that the proper functioning of the minor spliceosome is critical for early animal development.

Our *zrsr2*-knockout zebrafish model allowed us to investigate the role of *ZRSR2* during vertebrate embryonic hematopoiesis, as the previous studies in mice were inconclusive due to the compensation by its functional paralog, *Zrsr1* [23,24]. *zrsr2*-knockout zebrafish embryos displayed a lack of HSCs in the CHT region at 3 dpf and reduced numbers of cells from the erythroid and myeloid lineages at 5 dpf, suggesting that a functional Zrsr2 is required for normal hematopoietic development. However, we did not observe significant dysregulation of the genes involved in the hematopoietic pathway via RNA-Seq. This is probably due to our approach of using the whole embryo (minus the heads) for RNA-Seq.

Our study also demonstrated that the critical role of Zrsr2 in the regulation of the expression of genes with U12-type introns as a component of the minor spliceosome is conserved in zebrafish. Similar to human MDS bone marrow samples from patients with *ZRSR2* mutations, *zrsr2*-deficient zebrafish showed preferential retention of more than one-third of all U12-type introns (218/662). Specifically, the *e2f* and *mapk* gene families seem to be most affected by retained introns when the minor spliceosome function is disrupted [19,20,24,35,39]. Among the genes with retained introns in *zrsr2^hg129/hg129^* mutants, we also identified multiple genes from both the *e2f* and *mapk* gene families (*e2f4, mapk3, mapk8a, mapk12a, mapk14a*). A recent study has provided strong evidence for a link between the retention of a specific U12-type intron in *LZTR1* and the pathogenesis of MDS [21]. Therefore, we checked our list of genes with differential IR events and confirmed that this U12-type intron in *lztr1* was conserved in zebrafish and was differentially retained in *zrsr2^hg129/hg129^* embryos (*p* < 0.0001) (Supplementary Table S4).

Furthermore, we found that the genes affected by IR in the absence of a functional Zrsr2 in zebrafish during embryonic development are important for the processing of RNA and proteins and protein transport. These findings suggest that Zrsr2 is important for the regulation of these biological processes during embryonic development in zebrafish. Thus, it is possible that the morphological and hematopoietic defects observed in *zrsr2*-knockout embryos are due to the indirect effect of the dysregulation of these important cellular functions in tissues that are actively growing and differentiating. In conclusion, our study of *zrsr2*-deficient zebrafish provides strong support for the critical role of Zrsr2 in the splicing of minor introns in RNA and protein processing genes that play a crucial role in multiple developmental processes during embryogenesis. Further studies using hematopoietic cells from our *zrsr2*-knockout model for RNA-Seq will lead to a better understanding of the role of Zrsr2 in hematological diseases.

## 4. Materials and Methods

### 4.1. Zebrafish Husbandry and Ethics Statement

Zebrafish (*Danio rerio*) of WT strain TAB5 maintained in our laboratory were used in this study. All experiments were performed in compliance with the National Institutes of Health guidelines for animal handling and research using an Animal Care and Use Committee-approved protocol (G-05-5 assigned to RS). Zebrafish husbandry and embryo staging were performed as previously described [40]. All embryos were raised in an incubator at 28.5 °C until 5–6 dpf. Larvae and adult zebrafish were housed in a recirculating aquatic system with a light/dark cycle (14/10 h, respectively) and a water temperature of 28 °C.

### 4.2. Generation of zrsr2 Mutants, Crispants and Genotyping

Two single-guide RNAs targeting exons 7 (5′–GGAGCATCTGGGTTTTTCCA–3′) and 8 (5′–TGCCAGTCTGGAGTACAGTG–3′) were designed and synthesized as described previously [41]. WT zebrafish embryos were injected with 50 pg of each single guide RNA mixed with 150 pg of Cas9 mRNA. Embryos injected with sgRNA to exon 7 were grown to adulthood for generation of *zrsr2* knockout zebrafish model. Founder screening, identification of heterozygous F1 progeny, and all subsequent genotyping during line maintenance and phenotype analysis were performed by fluorescent PCR, as described previously [41], using primers listed in Supplementary Table S1. For crispant analysis, injected embryos were either harvested and fixed at 3 dpf for WISH or observed for morphological phenotypes up to 5 dpf, and harvested for DNA extraction. Fluorescent PCR and CRISPR-STAT [42] were performed on all crispant embryos to correlate the observed phenotypes with the reduction of WT peak and the presence of indel alleles.

### 4.3. RNA Extraction, RT-PCR, qRT-PCR, and Sequencing

RT-PCR and qRT-PCR were used to determine the expression of both isoforms of *zrsr2* throughout embryonic development using primers described in Supplementary Table S1. RNA was extracted from WT embryos collected at 1 hpf, 4 hpf, 19 hpf, 24 hpf, 2 dpf, 3 dpf, and 5 dpf, using Direct-zol RNA Microprep kit (Zymo Research, Irvine, CA, USA). cDNA for RT-PCR was synthesized using SuperScript IV One-Step RT-PCR System (Thermo Fisher Scientific, Waltham, MA, USA). RT-PCR products were analyzed by gel electrophoresis using 1.5% agarose gels. cDNA for qRT-PCR was generated with iScript cDNA synthesis kit (Bio-Rad, Hercules, CA, USA). qPCR was then performed using Power SYBR Green PCR Master Mix (Applied Biosystems, Waltham, MA, USA) according to the manufacturer’s instruction, using *actb2* primers (Supplementary Table S1) as internal controls. RT-PCR with primers shared by isoforms 201 and 204 was used to confirm the presence of CRISPR/Cas9-induced deletion in RNA from 5 dpf, WT, and mutant embryos. These RT-PCR products were sequenced using Sanger sequencing as described previously [43].

### 4.4. Generation of zrsr2 Riboprobe, WISH, and o-Dianisidine Staining

To generate a template for *zrsr2* riboprobe synthesis for WISH, we performed RT-PCR using RNA from 5 dpf WT embryos and primers from the region shared by both isoforms (Supplementary Table S1). The RT-PCR product was cloned into a pCR4-TOPO vector (Thermo Fisher Scientific, Waltham, MA, USA) and sequence-verified by Sanger sequencing. The plasmid DNA was extracted using QIAprep Spin Miniprep Kit (Qiagen, Germantown, MD, USA), digested with *SpeI*, and labeled with a DIG labeling kit (Sigma-Aldrich, St. Louis, MO, USA) using T7 polymerase. Antisense riboprobes for all other WISH markers used in this study were also generated using a DIG labeling kit (Sigma-Aldrich, St. Louis, MO, USA). Z*rsr2^hg129/+^* fish were in-crossed, and embryos were collected at various developmental stages. Collected embryos were euthanized and fixed overnight in 4% PFA at 4 °C. WISH was performed as described previously [44]. For *o*-dianisidine staining, euthanized embryos were incubated in *o*-dianisidine (MP Biomedicals, Costa Mesa, CA, USA) solution for 15 min in the dark [45], followed by fixation in 4% PFA prior to imaging. Embryos used for WISH and *o*-dianisidine staining were genotyped via fluorescent PCR, as described previously [41], using REDExtract-N-Amp PCR ReadyMix (Sigma-Aldrich, St. Louis, MO, USA) for product amplification.

### 4.5. Alcian Blue Staining

Embryos from *zrsr2^hg129/+^* in-crosses were euthanized and collected at 4 dpf, fixed in 4% PFA for 2 h at room temperature, and then stained in 0.15% Alcian Blue (Sigma-Aldrich, St. Louis, MO, USA), 50% EtOH, 0.1M HCl (pH 1) overnight, as described previously [46]. Embryos were then rinsed in 100% EtOH and rehydrated in a series of three 5 min EtOH/1x PBS washes. Embryos were then digested in 0.05% trypsin dissolved in 35% saturated NaBO_4_ for 2.5 h, followed by bleaching in a 3% H_2_O_2_ 1% KOH solution. Embryos were stored in 70% glycerol at 4 °C, imaged, and genotyped via fluorescent PCR, as described above.

### 4.6. Imaging and Image Analysis

Images of embryos for morphological phenotypes, Alcian blue staining, *o*-dianisidine staining, and WISH were acquired with a Leica M205 microscope, Leica DFC7000G camera, and the Leica Application Suite X Imaging Software Suite version 3.4.1 (Leica Microsystems, Deerfield, IL, USA). FIJI imaging software version 2.1.0 was used to quantify the staining intensity for *o*-dianisidine signals, as described in [47]. Differences between *zrsr2^hg129/hg129^* and control embryos were determined using an unpaired *t*-test with Welch’s correction.

### 4.7. RNA Extraction and Sequencing (RNA-Seq)

*zrsr2^hg129/+^* and WT sibling adult fish were in-crossed to collect mutant and WT embryos respectively. At 3 dpf, embryos from each clutch were euthanized with MS-222 (0.4 g/L) and decapitated to collect the heads for genotyping to identify homozygous mutant embryos. The embryo bodies were preserved in DNA/RNA Shield (Zymo Research, Irvine, CA, USA) in a corresponding 96-well plate for RNA extraction. After genotyping, 3 pools of 10 preserved embryos were collected from mutant and WT crosses. The pooled embryos were homogenized with a glass homogenizer, and the RNA was extracted using miRNeasy kit (Qiagen, Germantown, MD, USA) according to manufacturer’s instructions. RNA quantity and integrity were checked by the Bioanalyzer (Agilent, Santa Clara, CA, USA). The RNA Integrity number (RIN) values for samples used for RNA-Seq were as follows: WT triplicates (9.7, 9.7, 9.2), Mutant triplicates (9, 8.9, 9). An amount of 500 nanograms of total RNA was used in conjunction with the TruSeq^®^ Stranded Total RNA Library Prep kit (Illumina, San Diego, CA, USA). The libraries were checked for quality via the Bioanalyzer and quantitated by the Qubit (Thermo Fisher Scientific, Waltham, MA, USA). Equimolar amounts of each library were pooled and run on an Illumina Next-Seq 550/500 High Output Kit v2.5 (300 cycles). All RNA-Seq data are available at GEO (https://www.ncbi.nlm.nih.gov/geo/ accessed on 10 January 2022) under the accession number: GSE193365.

### 4.8. Bioinformatics Analysis of RNA-Seq Data

Low quality reads, identified by having N-base ratios above 10% or low-quality bases ratio above 50%, and reads containing adapter sequences were removed. The clean reads were aligned to GRCz11 with hisat2 (2.2.1.0) [48] and sorted with sambamba (0.7.1) [49]. Reads were counted with Stringtie (2.0.3) [50] based on GRCz11.99 annotation. Differentially expressed genes were identified using DESeq2 [33], using 0.05 and 1 as the cutoff for *p*-adjusted and absolute Log_2_FoldChange values, respectively. A heatmap was generated with Heatmapper [51], using Average Linkage clustering and Pearson distance measurement. IRFinder was used to identify the introns that were differentially spliced between WT and mutant transcripts [36], and intron types were classified using the Intron Annotation and Orthology Database [7]. Enriched pathways associated with differentially expressed genes, and differentially spliced genes between WT and *zrsr2^hg129/hg129^* samples were identified in Metascape [52], which uses hypergeometric testing and a Benjamini-Hochberg *p*-value correction algorithm to identify statistically significant pathways. Comparison of U12-type (retained versus non-retained) introns was performed by extracting the sequences for each group and an analysis of their size distribution, base content, and terminal dinucleotides. For the analysis of the splice sites, the first and last 20 bases of all introns were extracted and illustrated with WebLogo [53].

## Data Availability

All RNA-Seq data are available at GEO (https://www.ncbi.nlm.nih.gov/geo/ accessed on 10 January 2022) under the accession number: GSE193365.

## Supplementary Materials

The following supporting information can be downloaded at: https://www.mdpi.com/article/10.3390/ijms231810668/s1.
