# Supplementary material for: Zrsr2 Is Essential for the Embryonic Development and Splicing of Minor Introns in RNA and Protein Processing Genes in Zebrafish
Source: Int J Mol Sci. 2022 Sep 14;23(18):10668. doi: 10.3390/ijms231810668 (PMC9501576; doi:10.3390/ijms231810668)

Supplementary Figure S1. Alignment of human ZRSR2 with both isoforms of zebrafish Zrsr2. Sequence alignment was performed using CLUSTALW in the MacVector software. Identical amino acids are shaded dark grey and similar amino acids are shaded light grey. Numbers on the left and right sides of each row represent the amino acid numbers in the protein sequence. The green boxes indicate the zinc finger domains, and the purple box indicates the RNA recognition motif.

```
ZRSR2_Human          1                  MAAPEKMTFPEKPSHKKYRAALKKEKRKKRRQELARLRDSGLS  43
Zrsr2_Zebrafish_201  1  MTEKGCCSIQTETQDSENRLNMCETPSVVLSHKKRRAALKRERRKRKRQALAKIKEADNN  60
Zrsr2_Zebrafish_204

ZRSR2_Human         44  QKEEEEDTFIEEQQLEEEKLLERERQRLHEEWLLREQKAQEEFRIKKEKEEAAKKRQEEQ  103
Zrsr2_Zebrafish_201 61  ---LQNEELQSEAEDEDKLKVDEESERLHQEWMERERIAQEEFRLKKEREDAAQQRKEEE  117
Zrsr2_Zebrafish_204

ZRSR2_Human        104  ERKLKEQWEEQQRKEREEEEQKRQEKKEKEEALQKMLDQAENELENGTTWQNPEPPVDFR  163
Zrsr2_Zebrafish_201 118 ERKIKEEWEEQQRKDKEEREHKEQEKREREEAVQKMLDQAESQLENGGPWKNPDAPKDYG  177
Zrsr2_Zebrafish_204  1                                     MLDQAESQLENGGPWKNPDAPKDYG  25

ZRSR2_Human        164  VMEKDRANCPFYSKTGACRFGDRCSRKHNFPTSSPTLLIKSMFTTFGMEQCRRDDYDPDA  223
Zrsr2_Zebrafish_201 178 -TEKDKANCPFFLKTGACRFGDRCSRKHDHPASSCTLMVRGMFVSFGMEQSRRDDYDTDA  236
Zrsr2_Zebrafish_204 26  -TEKDKANCPFFLKTGACRFGDRCSRKHDHPASSCTLMVRGMFVSFGMEQSRRDDYDTDA  84

ZRSR2_Human        224  SLEYSEEETYQQFLDFYEDVLPEFKNVGKVIQFKVSCNLEPHLRGNVYVQYQSEEECQAA  283
Zrsr2_Zebrafish_201 237 SLEYSEEELHQQFLDFYEDALPEFKNAGRVVQFKVSCNFEPHLRGNVYVQYETEEQCKEA  296
Zrsr2_Zebrafish_204 85  SLEYSEEELHQQFLDFYEDALPEFKNAGRVVQFKVSCNFEPHLRGNVYVQYETEEQCKEA  144

ZRSR2_Human        284  LSLFNGRWYAGRQLQCEFCPVTRWKMAICGLFEIQQCPRGKHCNFLHVFRNPNNEFWEAN  343
Zrsr2_Zebrafish_201 297 FVMFNGRWYAGRQLQCEFSPVTRWKTAICGLFDRRKCPKGKHCNFLHVFRNPDNEFWEAD  356
Zrsr2_Zebrafish_204 145 FVMFNGRWYAGRQLQCEFSPVTRWKTAICGLFDRRKCPKGKHCNFLHVFRNPDNEFWEAD  204

ZRSR2_Human        344  RDIYLSPDRTGSSFGKNSERRE----RMGHHDDYYSRLRG-RRNPSPD-HSYK--RNGES  395
Zrsr2_Zebrafish_201 357 RDLHMSPDRGGGFSARHMSGRDGMWSQRGNSSERFQRRRTDRRSHSRERYSYRGRRSRSR  416
Zrsr2_Zebrafish_204 205 RDLHMSPDRGGGFSARHMSGRDGMWSQRGNSSERFQRRRTDRRSHSRERYSYRGRRSRSR  264

ZRSR2_Human        396  ERKS-SRHRGKKSHKRTSKSRERHNSRSRGRNRDRSRDRSRGRGSRSRSRSRSRRSRRSR  454
Zrsr2_Zebrafish_201 417 ERRSRSRERRSRSRERRSRSRERQNRNRERQSRSRSREKRKSENSIRRSTSQREQSRRSR  476
Zrsr2_Zebrafish_204 265 ERRSRSRERRSRSRERRSRSRERQNRNRERQSRSRSREKRKSENSIRRSTSQREQSRRSR  324

ZRSR2_Human        455  SQSSSRSRSR-------GRRRSGNRD----------------RTVQSP------------  479
Zrsr2_Zebrafish_201 477 SRSQSRDRLKQSDSADSGKKMSKSNSPEHVSKNGSVKRKSHRKESQSPENTDRMDAHHHK  536
Zrsr2_Zebrafish_204 325 SRSQSRDRLKQSDSADSGKKMSKSNSPEHVSKNGSVKRKSHRKESQSPENTDRMDAHHHK  384

ZRSR2_Human        480  ---KSK*                                                       483
Zrsr2_Zebrafish_201 537 HSKKSKKKKKGKKKHKRKKSKSRSSSSSAGSDEESRIEGFEETPSKSQASVEECPAAASS  596
Zrsr2_Zebrafish_204 385 HSKKSKKKKKGKKKHKRKKSKSRSSSSSAGSDEESRIEGFEETPSKSQASVEECPAAASS  444

ZRSR2_Human
Zrsr2_Zebrafish_201 597 TGSPEAKGEATALDCCNPEEEMQSTVIDALKSDSITPSE*  636
Zrsr2_Zebrafish_204 445 TGSPEAKGEATALDCCNPEEEMQSTVIDALKSDSITPSE*  484
```

Supplementary Figure S2. RT-PCR data showing *zrsr2* expression at various stages of development. Agarose gel images showing RT-PCR products with primers unique to isoform-201 (418 bp product), unique to isoform 204 (514 bp product), common to both isoforms (697 bp product) and loading control *actb2* (232 bp product) in WT embryos at various stages of development as marked across the top. Water was used as a no template control (NTC).

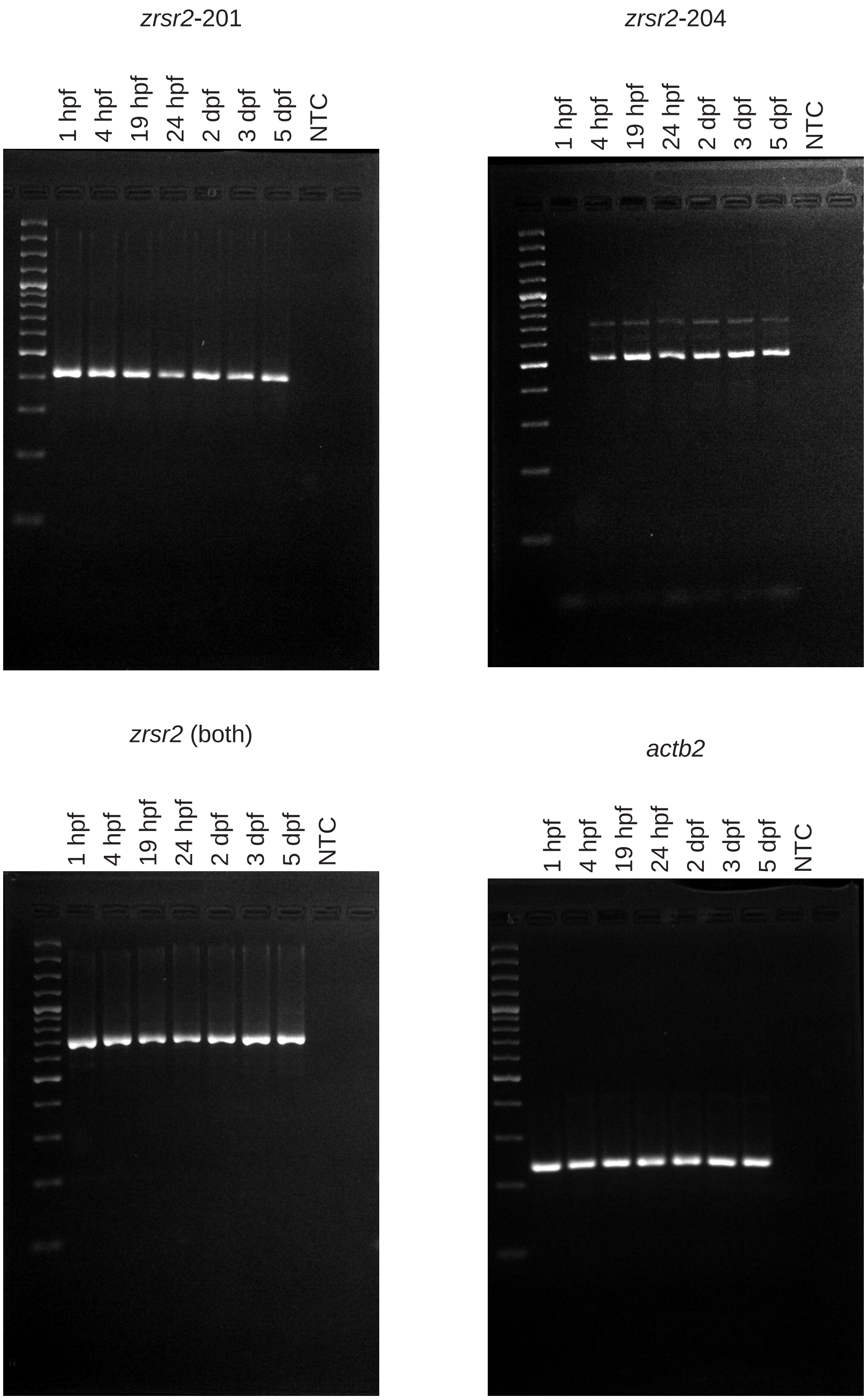

Supplementary Figure S3. Expression of mutant allele and normal expression of markers of early embryonic development in *zrsr2*$^{hg129/hg129}$ embryos. A) Sequence chromatograms showing the expression of the 11 bp deletion in cDNA from 5 dpf mutant embryos compared to the WT embryos. The black rectangle in WT sequence marks the nucleotides that are deleted in the mutant fish. B) WISH of *zrsr2*$^{hg129/hg129}$ (right panel) and control embryos (left panel) with *kdrl*, *myod1*, and *shh* at 24 hpf. Lateral views of embryos are shown in all images.

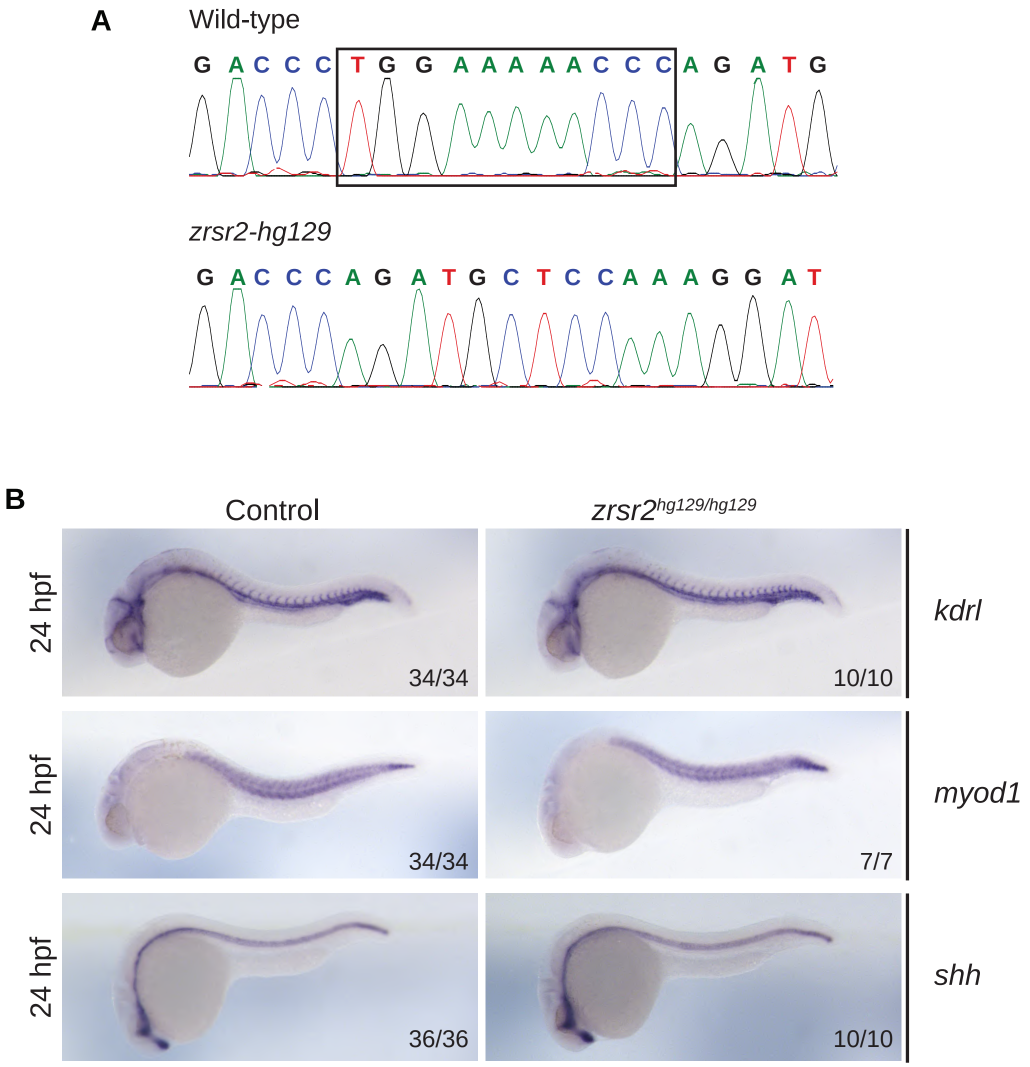

Supplementary Figure S4. ***zrsr2**$^{hg129/hg129}$* **embryos display normal primitive hematopoiesis and mild anemia.** A-C) WISH of *zrsr2*$^{hg129/hg129}$ (right panel) and control embryos (left panel) with primitive hematopoietic markers, *gata1*, *scl*, *mpo*, and *l-plastin* at 14 hpf and 20 hpf (*gata1*), 14 hpf and 22 hpf (*scl*), and 24 hpf (*mpo* and *l-plastin*). D) Ventral views of the yolk sac region and lateral views of the tail of 2 dpf *zrsr2*$^{hg129/hg129}$ embryos (bottom panel) compared to their siblings (top panel) stained with o-dianisidine showing reduced red blood cells in the mutant embryos. E) Quantitative image analysis of all stained embryos, measured in mean pixel intensity, showing the significantly reduced o-dianisidine staining ($p = 0.0015$) in *zrsr2*$^{hg129/hg129}$ embryos compared to their siblings. Horizontal bars indicate the mean and standard deviation of mean pixel intensity.

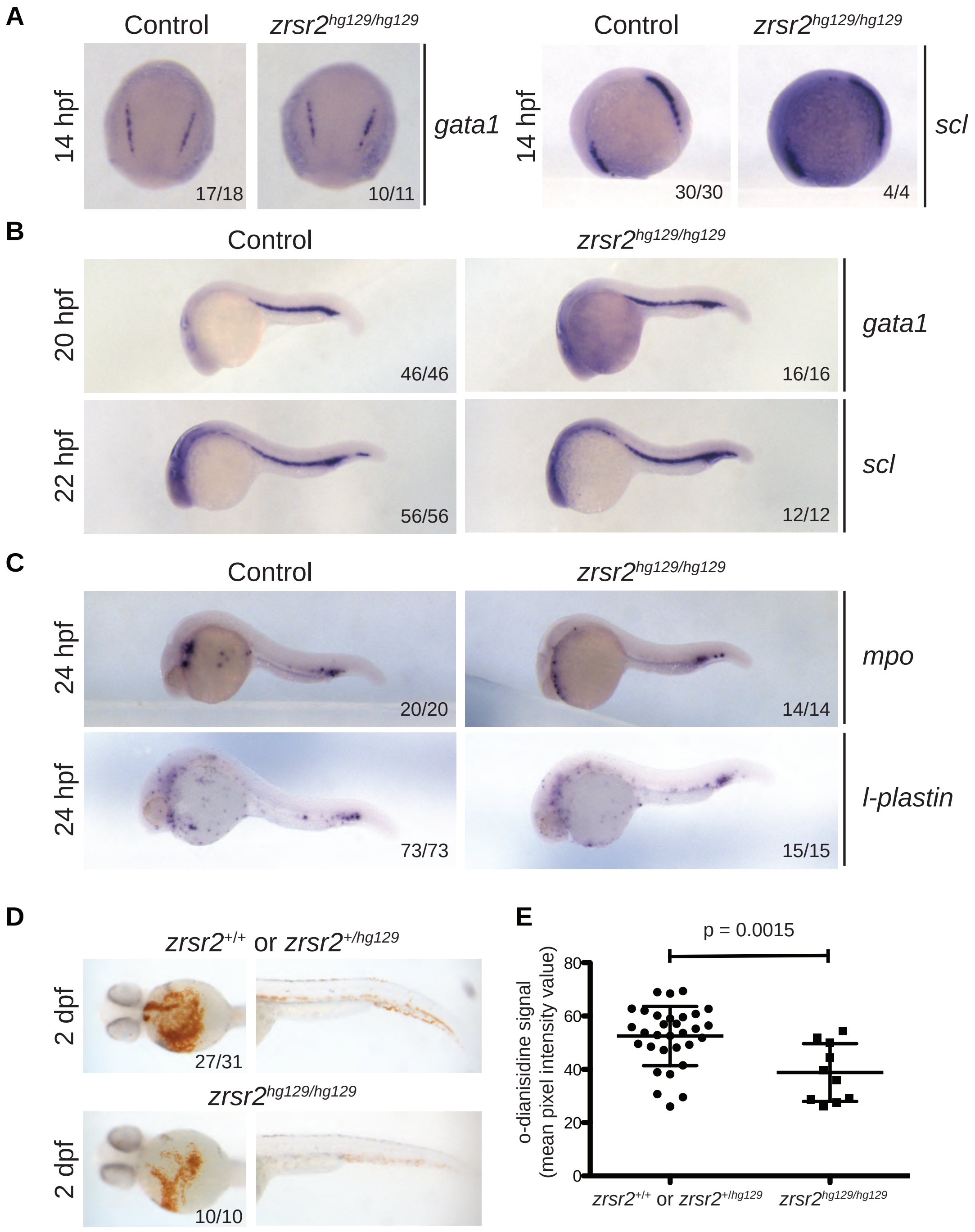

Supplementary Figure S5. *zrsr2*$^{hg129/hg129}$ embryos display normal specification of HSCs and early definitive hematopoiesis. WISH of *zrsr2*$^{hg129/hg129}$ (right panel) and control embryos (left panel) with HSC markers, *runx1* at 24 hpf and 36 hpf and *c-myb* at 2 dpf. Lateral views of embryos are shown in all images.

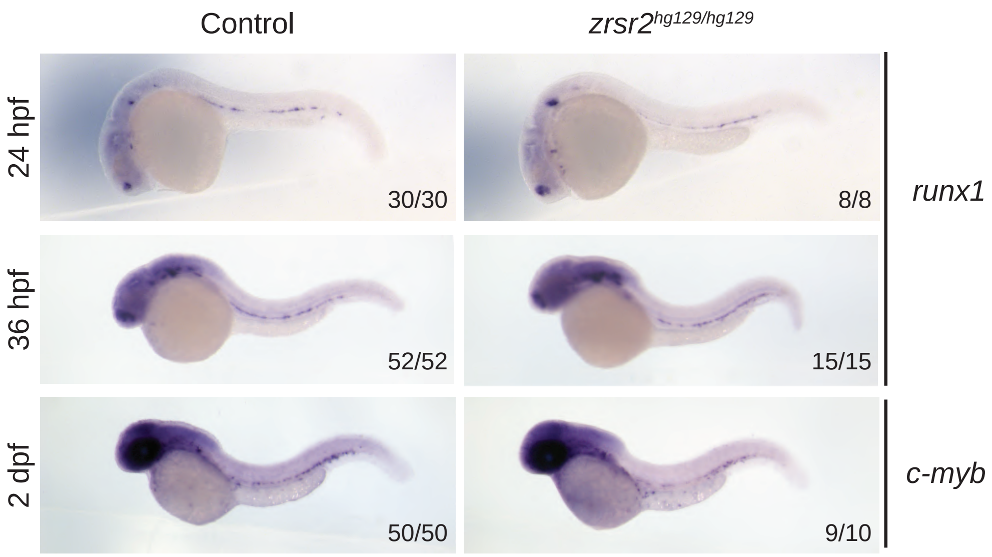

Supplementary Figure S6: *zrsr2* crispants phenocopy the *zrsr2*$^{hg129/hg129}$ genetic mutant. A) 5 dpf embryos injected with the sgRNAs targeting exon 7 (left panel) and exon 8 (right panel) depicting 3 classes of the morphological phenotypes based on the sgRNA activity. Class I: Lack of an inflated swim bladder; Class II: lack of an inflated swim bladder and defective jaw; Class III: lack of an inflated swim bladder, defective jaw and cardiac edema. Representative lateral view images of embryos for each class are shown with their corresponding CRISPR-STAT plots. Class I to III progressively presented an increased degree of sgRNA activity, shown by a reduction of the WT allele (red arrow) and presence of additional alleles with different indels. B) WISH with *c-myb* probe of exon 7 and exon 8 crispants at 3 dpf compared to uninjected control embryos. Lateral views of embryos are shown with purple arrows marking the CHT region showing reduced *c-myb* expression in the majority of crispant embryos.

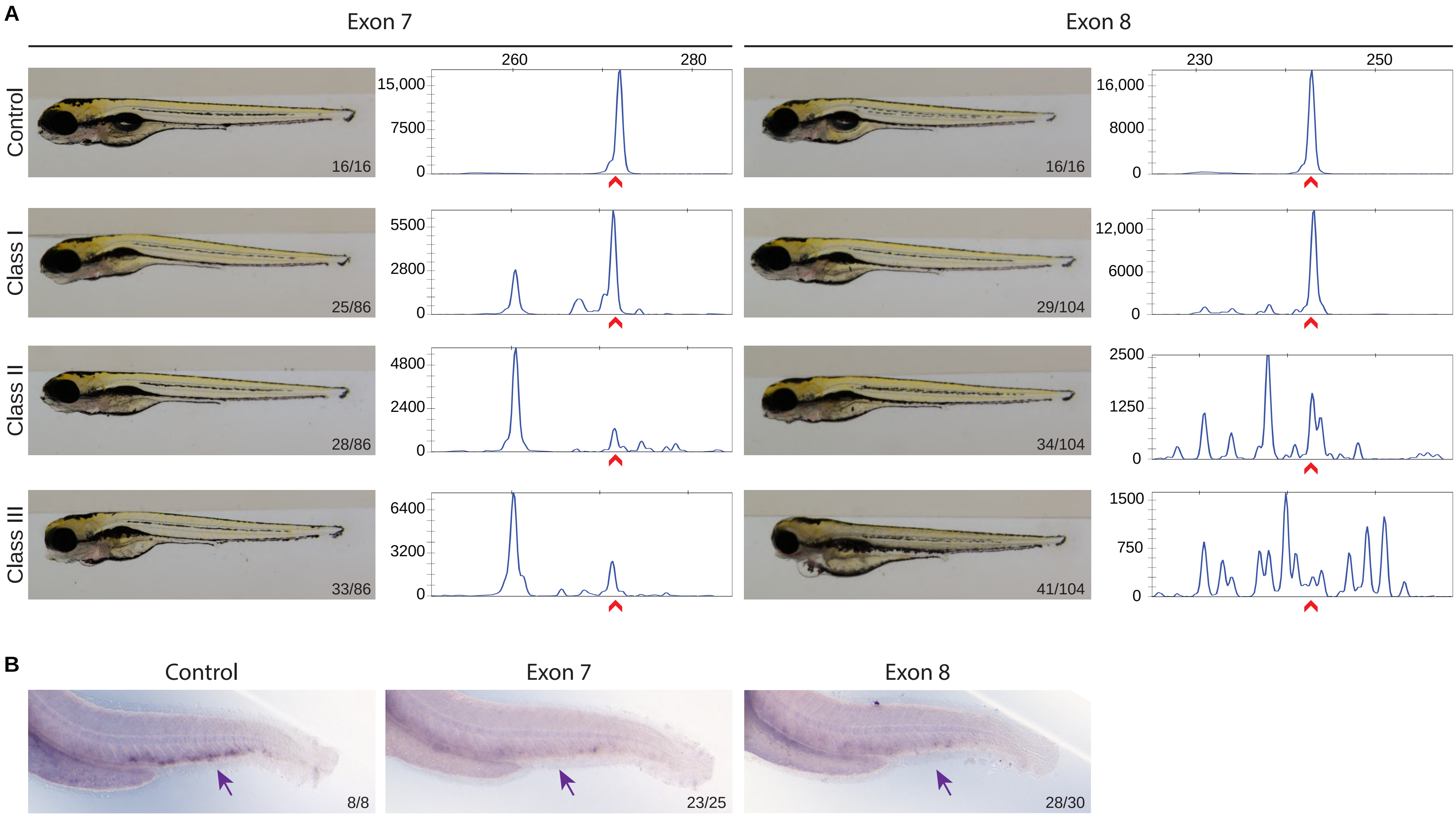

Supplementary Figure S7. Analysis of RNA-Seq data for quality and expression of *zrsr2* isoforms. A) Histogram of the distribution of raw read counts for the 3 WT and 3 *zrsr2*$^{hg129/hg129}$ samples. B) Percentages of genomic regions covered by the reads for each sample showing that majority of the reads lie within an exon. C) Expression of *zrsr2*-201 (left) and *zrsr2*-204 shown as fragments per kilobase of transcript per million mapped fragments (FPKM) on the Y-axis for each sample in WT (circle) and *zrsr2*$^{hg129/hg129}$ groups (triangle) on the X-axis. Red lines mark the average FPKM values within each group. (*: pval< 0.05; ns: not significant)

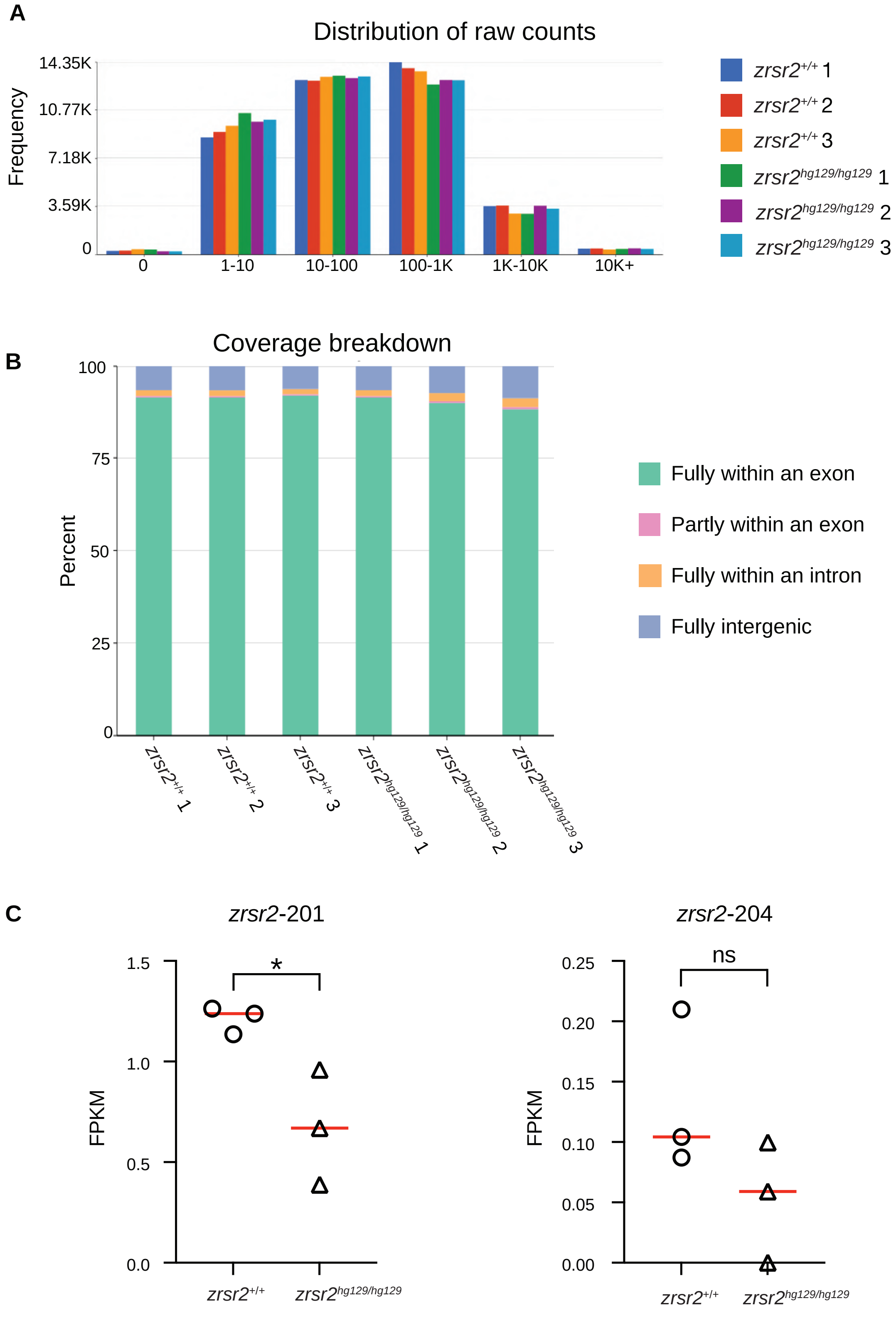

Supplementary Figure S8. Comparison of sequence features between retained versus not-retained U12-type introns in *zrsr2* mutants. Sequence features of 20 nucleotides from both ends of the intron sequences are shown as 5’ splice site and 3’ splice site in retained introns on the left and not-retained introns on the right side.

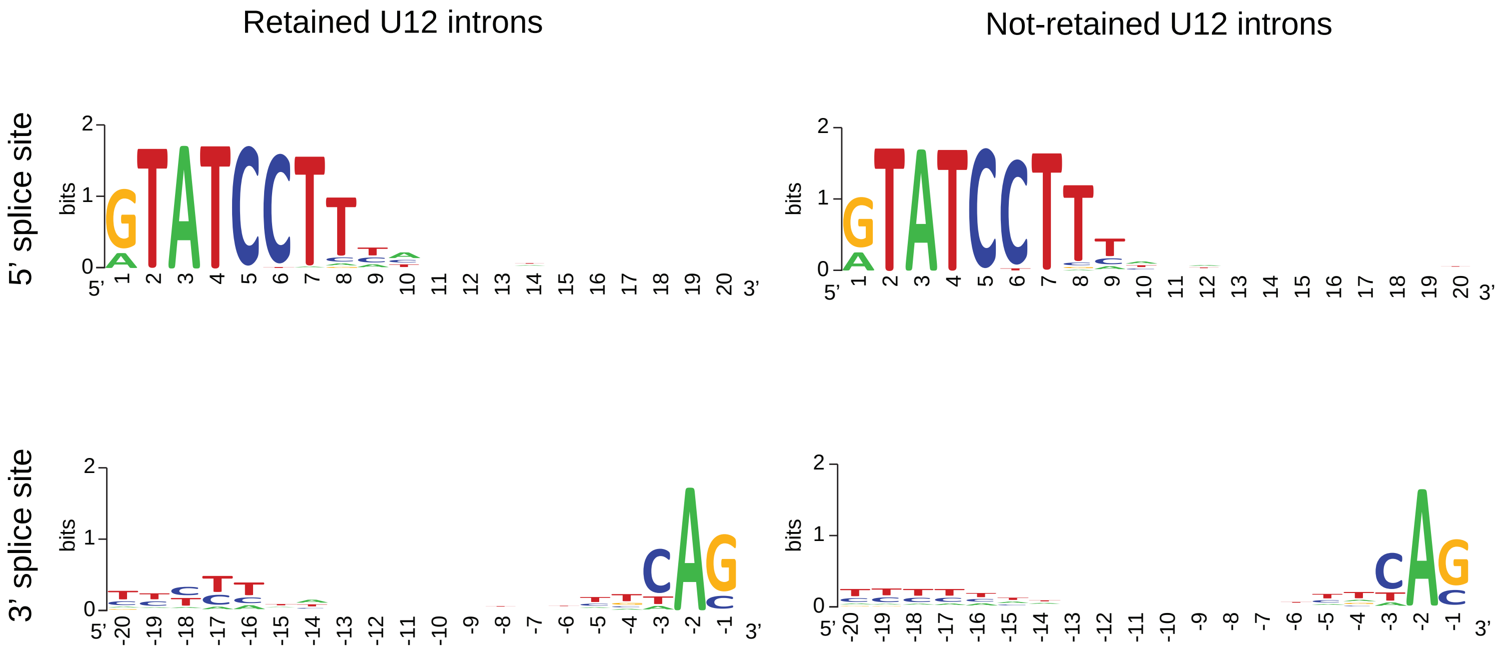

Supplement: Supplementary file 1 [file ijms-23-10668-s001.zip › ijms-1894501-Supplemental Figures.pdf]
